# Use of Antibody–Drug Conjugates in the Early Setting of Breast Cancer

**DOI:** 10.1177/11795549241260418

**Published:** 2024-06-17

**Authors:** Chrysanthi Koukoutzeli, Dario Trapani, Liliana Ascione, Elias Kotteas, Antonio Marra, Carmen Criscitiello, Giuseppe Curigliano

**Affiliations:** 1 Division of New Drugs and Early Drug Development for Innovative Therapies, European Institute of Oncology, IRCCS, Milan, Italy; 2 Department of Oncology and Haemato-Oncology (DIPO), University of Milan, Milan, Italy; 3 Oncology Unit, 3rd Department of Internal Medicine, Sotiria General Hospital and Athens School of Medicine, Athens, Greece

**Keywords:** Antibody–drug conjugate, ado-trastuzumab emtansine, trastuzumab deruxtecan, sacituzumab govitecan, early-setting breast cancer, adjuvant therapy, neoadjuvant therapy

## Abstract

Antibody–drug conjugates (ADCs) are anticancer agents with the capacity to selectively deliver their payloads to cancer cells. Antibody–drug conjugates consist of a monoclonal antibody backbone connected by a linker to cytotoxic payloads. Antibody–drug conjugate effect occurs either by directly targeting cancer cells via membrane antigen or through “bystander effect.” Antibody–drug conjugates have demonstrated efficacy against various types of tumors, including breast cancer. Ado-trastuzumab emtansine is presently the only approved ADC for the treatment of breast cancer in the early setting, while several ADCs are now approved for metastatic breast cancer. Due to the transformative impact that several ADCs have reported in the setting of advanced breast cancer, researchers are now testing more of such compounds in the early setting, to portend benefits to patients through highly potent anticancer drugs. Ongoing trials hold the potential to transform treatment protocols for early breast cancer in the near future. These trials are aiming at evaluating different treatment modulation approaches, as informed by breast cancer risk of recurrence, including toward treatment de-escalation. Efforts are provided in ongoing clinical trials to identify the patients who will benefit most, to pursue paradigms of precision medicine with the novel ADCs. This review focuses on the potential role of ADCs in early breast cancer, providing an overview of the latest progress in their development and how they are implemented in ongoing clinical trials.

## Introduction

The German physician and scientist Paul Ehrlich formulated the pharmacodynamic doctrine based on the assumption that pharmacological agents need to bind to a target to express their therapeutic properties, as synthetized in his statement “we have to learn how to aim chemically.” This concept led Ehrlich in the 1900s to the idea of a “magic bullet” to target antimicrobial agents with specific drugs for the treatment of human diseases.^1,2^ Currently, such a therapeutic paradigm aligns with the mechanism of action of antibody–drug conjugates (ADCs). Antibody–drug conjugates are a class of drugs with an antibody backbone attached to chemotherapeutic payloads. The integration of chemotherapeutic and targeted agents in this combination enables the precise and efficient delivery of cytotoxic agents. Antibody–drug conjugates consist of a monoclonal antibody (mAb) backbone linked to cytotoxic payloads, and are able to exert their therapeutic effect on cells expressing specific target antigens that are recognized by the mAb.^3,4^ Therefore, ADC can target target cells like “Trojan horses,” then crossing the cell membrane to deliver the cytotoxic effect directly and, in the case of certain new ADCs, affecting nearby tissues through a ‘‘bystander effect” which can maximize the effectiveness while potentially minimizing the overall toxicity.^5,6^

Antibody–drug conjugates are rapidly expanding in the field of modern oncology. They have been proven effective in treating metastatic breast cancer and they are already showing promising results in the early stages of the disease. So far, a total of seven ADCs have been granted approval for the treatment of solid tumors, globally, of which four for breast cancer (Table 1). This review focuses on the role of ADCs in breast cancer and provides an overview of the current advancements in their development for the early treatment of breast cancer (Table 2). Finally, it investigates potential approaches to improve the effectiveness of ADCs and overcome resistance.

**Table 1.** Summary of approved antibody–drug conjugates in solid tumors.

Drugs (trade name)/(company)	Target antigens	Linkers	Payloads	Average DAR	Approved countries	Approved date	Approved indications
Ado-trastuzumab emtansine (Kadcyla®)/(Roche)	HER2	SMCC	DM1	3.5	FDA/EMA/PMDA/NMPA	February 22, 2013	Adjuvant treatment of patients with HER2-positive early breast cancer who have residual invasive disease after neoadjuvant taxane and trastuzumab-based therapy
Enfortumab vedotin (Padcev®)/(Seagen)	Nectin-4	mc-VC-PABC	MMAE	3.8	FDA	December 18, 2019	Locally advanced or metastatic urothelial cancer who have had prior platinum chemotherapy and a PD-L1/PD-1 inhibitor
Fam-trastuzumab deruxtecan (Enhertu®)/(Daiichi Sankyo)	HER2	Tetrapeptide	DXd	7-8	FDA/EMA/PMDA	December 20, 2019	Adult patients with unresectable or metastatic HER2-positive breast cancer who have received two or more prior anti-HER2-based therapies in the metastatic setting; locally advanced or metastatic HER2-positive gastric or gastroesophageal junction adenocarcinoma who have received a prior trastuzumab-based therapy
Sacituzumab govitecan (Trodelvy®)/(Immunomedics)	Trop-2	CL2A	SN38	7.6	FDA	April 22, 2020	Patients with unresectable locally advanced or metastatic TNBC who have received two or more prior systemic therapies, including at least one for metastatic disease
Cetuximab sarotalocan (Akalux®)/(Rakuten Medical)	EGFR	NA	IRDye700DX	1.3-3.8	PMDA	September 25, 2020	Unresectable locally advanced or recurrent HNSCC
Disitamab vedotin (Aidixi®)/(RemeGen)	HER2	mc-VC-PABC	MMAE	4	NMPA	June 8, 2021	Patients with locally advanced or metastatic gastric cancer (including gastroesophageal junction adenocarcinoma) who have received at least two types of systemic chemotherapy
Tisotumab vedotin (Tivdak®)/(Genmab/Seagen)	TF	mc-VC-PABC	MMAE	4	FDA	September 20, 2021	Adult patients with recurrent or metastatic cervical cancer whose disease has progressed on or after chemotherapy

Abbreviations: CL2A, a cleavable complicated PEG8- and triazole-containing PABC-peptide-mc linker; DAR, drug-to-antibody ratio; DM1, derivative of maytansine 1; DXd, exatecan derivative for ADC; EGFR, epidermal growth factor receptor; EMA, European Medicines Agency; FDA, Food and Drug Administration; HER2, human epidermal growth factor receptor 2; HNSCC, head and neck squamous cell carcinoma; mc-VC-PABC, maleimidocaproyl-valine-citrulline-p-aminobenzoyloxycarbonyl; MMAE, monomethyl auristatin E; NMPA, National Medical Products Administration of China; PD-1, programmed cell death protein-1; PD-L1, programmed cell death-ligand 1; PMDA, Pharmaceuticals and Medical Devices Agency of Japan; SMCC, succinimidyl-4-(N-maleimidomethyl)cyclohexane-1-carboxylate; SN38, active metabolite of irinotecan; TF, tissue factor; TNBC, triple-negative breast cancer.

**Table 2.** Ongoing clinical trials with antibody–drug conjugates in the early setting of breast cancer.

ADC investigated as single agents									
Drugs (trade name)		Target Target antigens	Linkers	Payloads	Average DAR	Setting	Phase of drug development	ClinicalTrials.gov identifier	Study description
Fam-trastuzumab deruxtecan (Enhertu®)		HER2	Tetrapeptide	DXd	7-8	Post-neoadjuvant	3	NCT04622319 (DESTINY-Breast05)	This study will evaluate the efficacy and safety of T-DXd compared to T-DM1 in HRP with residual invasive breast cancer following NT
						Neoadjuvant	2	NCT05704829 (ADAPTHER2-IV)	A superiority trial to demonstrate higher pCR rates in L-IRP HER2+ EBC. In addition, it aims to demonstrate excellent survival in patients treated with T-DXd (with the use of standard chemotherapy restricted only to patients with substantial residual tumor burden after T-DXd treatment).
						Neoadjuvant	3	NCT05113251 (DESTINY-Breast11)	This study will evaluate the efficacy and safety of T-DXd in the NS, in high-risk, HER2-positive EBC. Participants will be randomized to one of three arms: T-DXd monotherapy (arm A), T-DXd followed by THP (arm B), or ddAC-THP (arm C).
						Neoadjuvant	2	NCT05710666 (SHAMROCK)	Patients with EBC HER2+ breast cancer will receive NT with T-DXd for up to six cycles. Primary outcome: The percentage of patients who achieve a pCR following T-DXd treatment.
						Neoadjuvant	2	NCT05900206 (ARIADNE)	A study to compare T-DXd to standard preoperative treatment in patients with non-metastatic HER2-positive BC
Trastuzumab rezetecan		HER2		SHR9265	5.7	Post-neoadjuvant	3	NCT06126640	This study aims to Active-Controlled Study of trastuzumab rezetecan T-DM1 in HER2-positive primary BC participants with RID following NT
						Neoadjuvant	2	NCT05911958	A study to evaluate the efficacy and safety of trastuzumab rezetecan for early-stage or locally advanced BC patients with HR-positive, low HER2 expression
Disitamab vedotin (Aidixi®)		HER2	mc-VC-PABC	MMAE	4	Neoadjuvant	2	NCT05134519	This is a single-arm exploratory study to evaluate the effect of RC48 in HER2+ NT and to assess the non-inferiority of RC48
Trastuzumab duocarmazine		HER2	Cleavable-VC	seco-DUBA	2.8	Neoadjuvant	2	NCT01042379 (I-SPY)	An investigational treatment arm for patients with HER2-positive and HER2-low early breast cancer
ARX788		HER2	pAcF	MMAF	1.9	Neoadjuvant	2	NCT01042379 (I-SPY2)	Personalized adaptive treatment in HER2-positive early BC
TQB2102		HER2	Enzyme-cleavable	TopI inhibitor		Neoadjuvant	2	NCT06198751	To evaluate the efficacy and safety of neoadjuvant treatment with TQB2102 for injection in patients with HER2-positive BC
Sacituzumab govitecan (Trodelvy®)		Trop-2	CL2A	SN38	7.6	Post-neoadjuvant	3	NCT04595565 (SASCIA)	HER2-BC with RD after NT with 1:1 allocation to: Arm A: SG; Arm B: TPC in patients with HR+ breast cancer, endocrine-based therapy, including the use of CDK4/6 inhibitors, will be administered according to local guidelines. Adjuvant pembrolizumab may be given prior to randomization until the completion of radiotherapy
ADC investigated as single agents									
Drugs (trade name)		Target antigens	Linkers	Payloads	Average DAR	Setting	Phase of drug development	ClinicalTrials.gov identifier	Study description
Patritumab deruxtecan		HER3	Tetrapeptide	DXd	4	Neoadjuvant	1	NCT04610528 (TOT-HER3)	A study to evaluate the biological effect of HER3-DXd in treatment-naïve patients with HR+/HER2-EBC, whose primary tumors are ⩾1 cm by ultrasound. Patients will be enrolled in four cohorts according to the mRNA-based ERBB3 expression by central assessment. The objective of the study is to evaluate the biological activity of HER3-DXd in patients with treatment-naïve EBC.
						Neoadjuvant	2	NCT05569811 (VALENTINE)	An exploratory study with primary operable HR+/HER2-negative breast cancer with ki67 ⩾20% and/or high genomic risk (defined by gene signature) to evaluate the clinical benefit and biological effects of HER3-DXd with/without letrozole as a NT
Ladiratuzumab vedotin		SLC39A6	mc-val-cit-PABC	MMAE	4	Neoadjuvant	1	NCT01042379 (I-SPY)	A study to evaluate the efficacy of NT with novel drugs in sequence with standard chemotherapy to identify treatment strategies for subsets based on molecular characteristics of their disease with a high estimated pCR rate.
ADC investigated in combination regimens									
Drugs (trade name)	Combination regimens	Target antigens	Linkers	Payloads	Average DAR	Setting	Phase of drug development	ClinicalTrials.gov identifier	Study description
Ado-trastuzumab emtansine (Kadcyla®)	Atezolizumab	HER2; PD-L1	SMCC	DM1	3.5	Post-neoadjuvant	3	NCT04873362 (ASTEFANIA)	A study to evaluate HER2+ primary breast cancer that has been treated with NT and HER2-directed therapy, including trastuzumab, followed by surgery, with evidence of RID in the breast and/or axillary lymph nodes
	Tucatinib	HER2; TKI	SMCC	DM1	3.5	Post-neoadjuvant	3	NCT04457596 (CompassHER2 RD)	A study to determine if iDFS with T-DM1 and tucatinib is superior to the iDFS in the control arm (T-DM1 + placebo) when administered to HRPs with HER2-positive breast cancer and RD after neoadjuvant HER2-directed therapy
	Trastuzumab; pertuzumab	HER2	SMCC	DM1	3.5	Post-neoadjuvant	2	NCT04733118 (PHERGAIN-2)	A study to evaluate the efficacy of a chemotherapy-free, pCR-guided strategy with trastuzumab and pertuzumab and T-DM1 in patients with previously untreated HER2+ EBC
Fam-trastuzumab deruxtecan (Enhertu®)	Anastrozole	HER2	Tetrapeptide	DXd	7-8	Neoadjuvant	2	NCT04553770 (TRIO-US B-12 TALENT)	This study is evaluating the effectiveness of T-DXd in treating HR+ cancer cells that express low levels of HER2, when given alone or in combination with anastrozole.
	Durvalumab (AstraZeneca)	HER2; PD-L1	Tetrapeptide	DXd	7-8	Neoadjuvant	2	NCT05795101 (TRUDI)	A study of neoadjuvant T-DXd plus durvalumab in patients with stage III, HER2+ or HER2-low IBC, who have not received prior treatment for ipsilateral breast cancer
Trastuzumab rezetecan	Pyrotinib maleate	HER2	Cleavable linker	SHR9265	5.7	Neoadjuvant	2	NCT05635487	A study to evaluate the efficacy and safety of trastuzumab rezetecan combined with pyrotinib maleate in stage II or III HER2-positive BC
Disitamab vedotin (Aidixi®)	Penpulimab	HER2; PD-1	mc-VC-PABC	MMAE	4	Neoadjuvant	2	NCT05726175	A study to evaluate the efficacy and safety of RC48 in combination with penpulimab as NT in patients with HER2-low EBC or locally advanced breast cancer
ADC investigated in combination regimens									
Drugs (trade name)	Combination regimens	Target antigens	Linkers	Payloads	Average DAR	Setting	Phase of drug development	ClinicalTrials.gov identifier	Study description
ARX788	Pyrotinib maleate	HER2	pAcF	MMAF	1.9	Neoadjuvant	2; 3	NCT05426486	A phase II or III neoadjuvant study comparing the efficacy and safety of ARX788 in combination with pyrotinib maleate vs TCbHP in patients with HER2+ BC
	Pyrotinib maleate	HER2	pAcF	MMAF	1.9	Neoadjuvant	2	NCT04983121	This trial observes the effectiveness and safety of pyrotinib maleate combined with ARX788 NT in stage II or III HER2-positive BC patients experiencing a poor efficacy of trastuzumab and pertuzumab
	Cemiplimab	HER2; PD-1	pAcF	MMAF	1.9	Neoadjuvant	2	NCT01042379 (I-SPY2)	Personalized adaptive treatment in HER2-positive early BC
Sacituzumab govitecan (Trodelvy®)	Atezolizumab Pembrolizumab	Trop-2; PD-L1	CL2A	SN38	7.6	Post-neoadjuvant	2	NCT04434040 (ASPRIA)	A study to determine if a combination of the two drugs works as a treatment for RD in the breast or lymph nodes and has circulating tumor DNA in the blood
		Trop-2; PD-1	CL2A	SN38	7.6	Post-neoadjuvant	3	NCT05633654 (ASCENT-05)	A study to determine if SG in combination with pembrolizumab given after surgery, is effective and safe compared to the TPC with either pembrolizumab or pembrolizumab plus capecitabine in participants with TNBC that still remains after surgery and NT
		Trop-2; PD-1	CL2A	SN38	7.6	Neoadjuvant	2	NCT04230109 (NeoSTAR)	A phase II, multi-arm, umbrella study of neoadjuvant SG-based therapy in patients with localized BC. The first cohort involves SG monotherapy. After completion of the monotherapy cohort, the combination therapy cohort (SG with pembrolizumab) will be open.
		Trop-2; PD-1	CL2A	SN38	7.6	Neoadjuvant	2	NCT05675579	A study to determine the efficacy of SG and pembrolizumab combination treatment on pCR/RCB-1 in the patients with early-stage TNBC who were resistant to the combination of immunochemotherapy
Datopotamab deruxtecan	Durvalumab	Trop-2; PD-L1	Tetrapeptide	DXd	4	Adjuvant	3	NCT05629585 (TROPION-Breast03)	This trial evaluates the efficacy of Dato-DXd with or without durvalumab when compared to ICT in patients with stage I to III TNBC with RD after NT

Abbreviations: BC, breast cancer; CDK4/6, cyclin-dependent kinase 4 and 6; CL2A, a cleavable complicated PEG8- and triazole-containing PABC-peptide-mc linker; DAR, drug-to-antibody ratio; Dato-DXd, datopotamab deruxtecan; ddAC-THP, dose dense doxorubicin plus cyclophosphamide followed by paclitaxel + trastuzumab pertuzumab; DM1, derivative of maytansine 1; DXd, exatecan derivative for ADC; EBC, early breast cancer; HER2, human epidermal growth factor receptor 2; HER2+, Human epidermal growth factor receptor 2 positive; HER3-DXd, patritumab deruxtecan; HR, hormone receptor; HRP, high-risk patients; IBC, inflammatory breast cancer; ICT, capecitabine and/or pembrolizumab; iDFS, invasive disease-free survival; L-IRP, low-intermediate risk patients; mc-val-cit-PABC, maleimidocaproyl-valyl-citrullinyl-p-aminobenzyloxycarbonyl; mc-VC-PABC, maleimidocaproyl-valine-citrulline-p-aminobenzoyloxycarbonyl; MMAE, monomethyl auristatin E; MMAF, monomethyl auristatin F; NS, neoadjuvant setting; NT, neoadjuvant therapy; pAcF, para-acetyl-phenylalanine; pCR, pathological complete response; PD-1, programmed cell death protein-1; PD-L1, programmed cell death-ligand 1; RC48, disitamab vedotin; RCB, residual cancer burden; RD, residual disease; RID, residual invasive disease; seco-DUBA, seco-duocarmycin-hydroxy-benzamide-azaindole; SG, sacituzumab govitecan-hziy; SLC39A6, zinc transporter LIV-1; SMCC, succinimidyl-4-(N-maleimidomethyl)cyclohexane-1-carboxylate; SN38, active metabolite of irinotecan; TCbHP, trastuzumab plus pertuzumab with docetaxel and carboplatin; T-DXd, trastuzumab deruxtecan; THP, paclitaxel-trastuzumab + pertuzumab; TKI, tyrosine kinase inhibitor; TNBC, triple-negative breast cancer ; TPC, treatment of physician’s choice; VC, valine-citrulline dipeptide.

## Structure of ADCs

Antibody–drug conjugates are composed of a mAb, capable to bind to the target cancer cells, and a payload, usually exerting cytotoxic properties. The mAb functions as the navigator guide of the ADCs toward target antigens that ideally should be predominantly expressed on the cancer cells or more broadly by cancer-associated components and to a lesser extent on healthy tissues, yielding minimal off-target toxicity.^7^ While ADCs usually are directed toward antigens on the cancer cells, some ADCs target stromal components: the stroma-directed ADCs appear to confer long-lasting anticancer responses, as the tumor microenvironment is capable of evolution through mutations under selective pressure in a less efficient way.^8^ Most of the mAbs at the backbone of ADCs belong to the immunoglobulin G (IgG) class. Among these, IgG1 has been favored, due to its longer serum half-life, stronger complement fixation, and Fcγ receptors binding, resulting in better anticancer activity.^9,10^ While broadly used, the use of large mAbs as backbone structures has recently been questioned due to their limited ability to penetrate tumor cells. Smaller format drug conjugates may offer a potential solution to yield better intra-tumor penetration, having better access to hidden epitopes otherwise inaccessible to larger antibodies. Ongoing studies are using antibodies derived from IgGs, small binding proteins, small molecules, and peptides such as antibody fragments, single-chain variable fragments, single-domains antibodies, and diabodies, to gain better penetration of ADCs a group of compounds that has been referred to as “anything–drug conjugates,” in literature.^11-13^ The linker molecule binds the mAb to the cytotoxic drug, having a key role in the definition of the stability of the drug. There are two main types of linkers in use: cleavable and non-cleavable linkers. Chemical or enzymatic triggers are employed by cleavable linkers to facilitate the targeted release of the cytotoxic payload at specific tumor sites. The stability of newly designed cleavable linkers in the bloodstream has been demonstrated.^4,14,15^ Non-cleavable linkers may also have plasma stability and therefore less off-target toxicity. Off-target toxicity can be observed when the payload is released in the plasma prior to delivery to the cancer cells.^16,17^ Linker molecules have been engineered nowadays through drug delivery optimization strategies as they are regulated by oxygen tension, pH, cancer-specific proteases, and lysosomal/vacuolar proteins that may be unique to the cancer cells and less likely to be expressed in non-cancer tissues. Moreover, ADCs having payloads with relatively high hydrophilicity could improve their pharmacokinetic parameters, and significantly decrease the nonspecific uptake and off-target toxicity. The high hydrophilicity may be able to mitigate the aggregation potential of the ADC with lipophilic payloads.^18^

The last element characterizing an ADC is the payload itself. The cytotoxic payload unleashes its effect after internalization of ADCs into cancer cells. In fact, payloads can diffuse locally, and exploit paracrine cytotoxicity, including bystander effects. They mostly belong to one of the three subgroups: tubulin inhibitors, DNA damaging/disrupting agents, and immunomodulators.^19^ The therapeutic effect of the ADCs can be a function of the amount and potency of the payloads they carry, commonly referred to as the drug-to-antibody ratio (DAR). The DAR is the varying number of the small molecule moiety (linker plus payload) attached to each antibody.^20^ The DAR varies between different ADCs and could determine the pharmacodynamic and pharmacokinetic profile. Of note, the hydrophobicity of the detached payload may be responsible for the “bystander effect,” as it is able to diffuse through the cellular bilayers, a phenomenon that allows the diffusion of cell-permeable cytotoxic agents into neighboring cells, therefore exerting cytotoxic effects in surrounding cells, regardless their antigen expression.^5,6^

**Figure 1.**
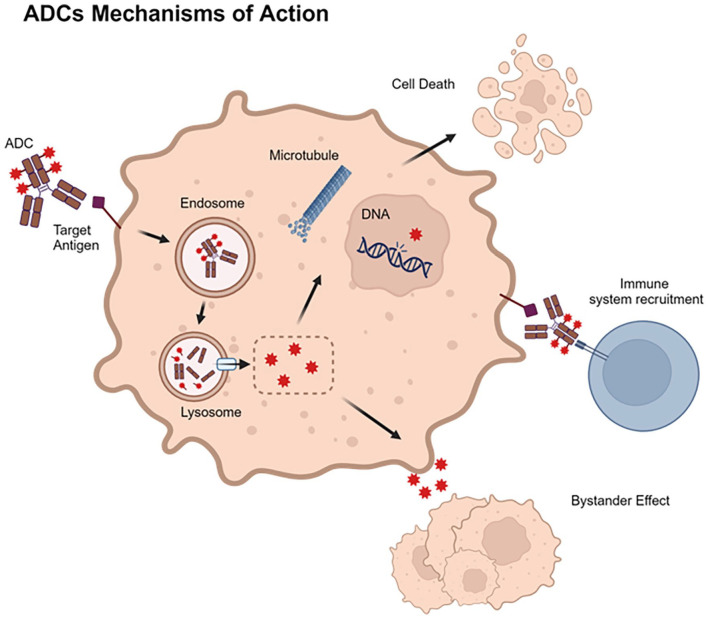
ADC mechanisms of action. ADC binds to the target antigen expressed on the cell membrane forming the ADC–antigen complex, which enters into the target cells via endocytosis. To complete ADC processing, the endosome fuses with the lysosome where ADCs are degraded, thus releasing their payloads. According to the specific cytotoxic moiety mechanism of action, each payload exerts its cytotoxic activity by damaging cell DNA, disrupting microtubules or via other mechanisms, causing cancer cells death. In addition, payloads that can cross cell membrane can also exert the so-called “bystander effect,” also allowing them to affect neighboring cells that do not express the target antigen. The presence of the ADC on target cell surface can also activate the immune system, promoting ADC anticancer activity. ADC indicates antibody–drug conjugate. Created with biorender.com (license, IEO).

Overall, ADCs bind to cancer cells via specific antigens and release payloads, resulting in tumor-specific and paracrine effects (Figure 1). Strategies for selecting the best target antigen, the most suitable cytotoxic drugs, the optimal linker, and the most appropriate payload have led to the development of state-of-the-art ADCs for a wide range of cancers, including breast cancer.

## ADCs in the Early Setting of Breast Cancer

**Figure 2.**
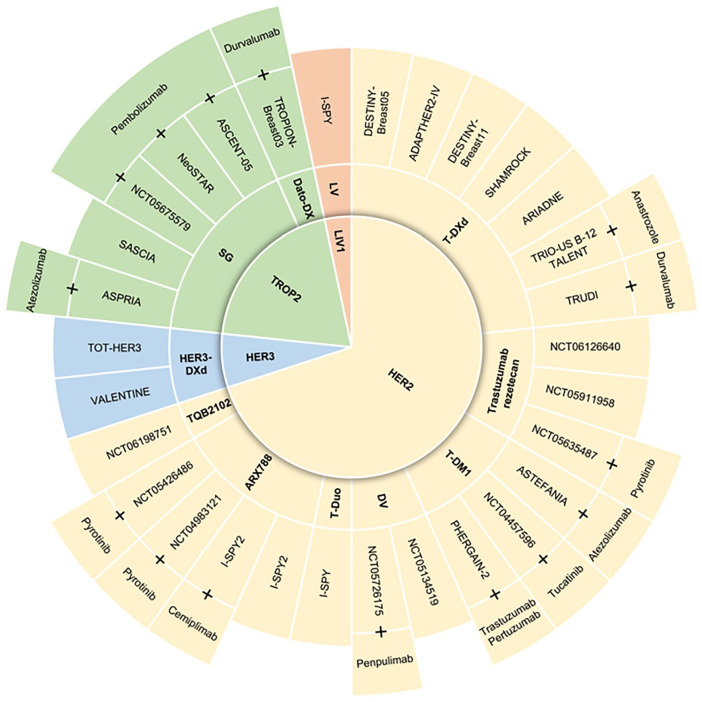
ADC and clinical trials in early breast cancer. Clinical trials with ADCs in the early setting of breast cancer. ADC indicates antibody–drug conjugate; Dato-DXd, datopotamab deruxtecan; DV, disitamab vedotin; HER2, human epidermal growth factor receptor 2; HER3, human epidermal growth factor receptor 3; HER3-DXd, patritumab deruxtecan; LIV1 or SLC39A6 or ZIP6, solute carrier family 39 member 6; LV, ladiratuzumab vedotin; SG, sacituzumab govitecan; T-DM1, ado-trastuzumab emtansine; T-DXd, trastuzumab deruxtecan; Trop-2, trophoblast cell surface antigen 2. Created with PowerPoint®.

Breast cancer has substantial inter- and intra-tumor heterogeneity. Heterogeneity can modulate the treatment effect of ADCs, especially when carrying non-diffusible payloads, and hamper the potential of ADCs in cancer treatment.^21^ Although breast cancer is predominantly detected in its early stages in Western countries, giving a significant number of patients the potential for successful treatment and cure, the prognosis for some early-stage tumors remains unfavorable, therefore requiring new agents and innovative strategies for the cure. Also, in the setting of highly curable cancer, the identification of similarly effective and less toxic treatments is desirable. As such, the use of ADCs in the early setting of care can be intended either as to intensify treatments in high-risk cancer or to provide less toxic alternatives to approved therapies. Several clinical trials are currently ongoing in the space of early breast cancer (Figure 2).

### Human Epidermal Growth Factor Receptor 2-targeting ADCs

The opportunity to target human epidermal growth factor receptor 2 (HER2) has led to the development of multiple targeted anticancer therapies directed against this target.^22^ Human epidermal growth factor receptor 2 has been the first cancer antigen toward which an ADC has been developed and approved (ado-trastuzumab emtansine [T-DM1]).^23^ Novel HER2-targeted mAbs have been engineered with the aim of improving function by binding the target with greater specificity or with the ability to bind additional epitopes, along with a plethora of ADCs with possible different pharmacological properties.^24^

#### Trastuzumab emtansine

It is an ADC derived from trastuzumab linked through a non-reducible thioether linkage (succinimidyl-4-(N-maleimidomethyl)cyclohexane-1-carboxylate) to a cytotoxic agent called emtansine (derivative of maytansine 1 [DM1]), a microtubule inhibitor derived from maytansine. Ado-trastuzumab emtansine enables intracellular delivery of DM1 to HER2-overexpressing cells.^25,26^

A decade ago, the HER2-targeted ADC T-DM1 was approved in the metastatic setting of HER2-positive breast cancer. This approval was based on the positive results of a randomized phase III clinical trial in patients who had previously received trastuzumab and taxane or relapsed during or within 6 months after completion of adjuvant therapy (EMILIA trial).^27^

In the adjuvant setting, the KATHERINE trial established T-DM1 as the standard of care for patients with HER2-positive early breast cancer who had residual invasive disease after completion of neoadjuvant standard treatments. After a median follow-up of 40 months, the trial demonstrated a statistically significant improvement in invasive disease-free survival (iDFS) in patients who received T-DM1 compared with those who received trastuzumab (hazard ratio 0.50; 95% CI: 0.39, 0.64; *P* < .0001). Distant recurrence as the first invasive disease event occurred in 10.5% of patients in the T-DM1 group and 15.9% of patients in the trastuzumab group.^28^ This was the first achievement in the context of early breast cancer with an ADC. Another study in the adjuvant setting, the ATEMP trial, aimed to determine whether treatment with T-DM1 had less toxicity than paclitaxel plus trastuzumab and provided a clinically acceptable iDFS in patients with stage I HER2-positive breast cancer.^29^ The development of TDM-1 as a neoadjuvant therapy has led to variable outcomes. First, the KRISTINE study revealed that the neoadjuvant systemic chemotherapy (docetaxel, carboplatin) plus dual HER2 blockade (trastuzumab plus pertuzumab) achieved a higher rate of pathological complete response (pCR) compared to T-DM1 plus pertuzumab (55.7% vs 44.4%).^30^ Subsequently, the Neopeaks study compared docetaxel + carboplatin + trastuzumab + pertuzumab (TCbHP) for six cycles or TCbHP for four cycles, followed by trastuzumab emtansine + pertuzumab (T-DM1 + P) for four cycles or T-DM1 + P for four cycles in patients with HER2-positive early primary breast cancer. The pCR rate with the standard TCbHP followed by the T-DM1 + P regimen was numerically higher than the TCbHP regimen alone, especially in patients with estrogen receptor-positive disease a result that may pave the way for a personalized treatment approach with ADCs.^31^ Furthermore, the WSG-ADAPT-TP trial demonstrated that patients who achieved pCR with the use of a de-escalated ADC-based neoadjuvant therapy, consisting of 12 weeks of T-DM1 with or without endocrine therapy (ET) or trastuzumab ± ET, had an excellent survival in hormone receptor (HR)+/HER2+ early breast cancer, without further adjuvant chemotherapy. Although, all trial arms had similar outcomes due to mandatory standard chemotherapy after non-pCR, the pCR rates were higher in the T-DM1 ± ET group compared with trastuzumab + ET. Moreover, the translational analysis in this study showed that the tumors with a high expression of immune markers, luminal A tumors (by PAM50), and PIK3CA wild-type tumors had a favorable prognosis with de-escalated therapy. This suggests that patients’ selection based on biomarkers or molecular subtypes may increase the efficacy of systemic de-escalated HER2-targeted approaches.^32^ In addition, the PHERGain-2 trial (NCT04733118) is testing mAb/ADC-based therapies with a neoadjuvant chemotherapy-free regimen consisting of trastuzumab and pertuzumab ± ET, followed by the same regimen or T-DM1 ± ET as determined by pCR after the surgery.^33^ Conventional chemotherapy is reserved for patients with disease progression after preoperative treatment.

Combinations of ADCs with other regimens, such as immunotherapy treatments, are also being investigated. There is increasing evidence that ADCs may enhance the efficacy of immunotherapeutic agents.^34^ Antibody–drug conjugates could act synergistically with immunotherapeutic drugs by stimulating dendritic cell maturation, T lymphocyte infiltration, immunogenic cell death, promoting immunological memory, and increasing immunoregulatory proteins such as programmed cell death-ligand (PD-L) 1 and major histocompatibility complex.^35-37^ ASTEFANIA (NCT04873362) is a phase III clinical trial that is evaluating the addition of the PD-L1 inhibitor atezolizumab to T-DM1 in high-risk HER2-positive patients with residual disease (RD) after preoperative treatment including taxane and trastuzumab. The primary endpoint is iDFS.^38^ Patients are being recruited regardless of their PD-L1 status. Similarly, the CompassHER2-RD trial is designed to improve the efficacy of T-DM1 when used in conjunction with tucatinib, a HER2-specific tyrosine kinase inhibitor (TKI), in the non-pCR setting following neoadjuvant HER2-directed therapy. This phase III trial is evaluating whether iDFS with T-DM1 plus tucatinib is superior to iDFS in the control arm with T-DM1.^39,40^

Ado-trastuzumab emtansine administration has been associated with a number of side effects, including thrombocytopenia, nausea, fatigue, diarrhea, and transaminitis. These adverse events (AEs) have been reported in approximately 40% of patients. Neuropathy may also occur in patients, particularly in the metastatic setting, when there is extended exposure to the drug.^41^ As such, novel clinical trials using T-DM1 in the (neo)adjuvant setting must take into account the safety profile to decide on the optimal treatment duration for ADCs.

#### Trastuzumab deruxtecan

Fam-trastuzumab deruxtecan-nxki (T-DXd) is a novel generation ADC constituted of a humanized anti-HER2 antibody, a tetrapeptide-based enzyme-cleavable linker and a topoisomerase I (TopI) inhibitor. Its DAR is equal to 8, which allows for the release of a high concentration of payload^42^ A high DAR can increase the efficacy but also can cause increase hydrophobicity and decrease solubility, resulting in poorer pharmacokinetics.^11^ The novel linker-payload system of T-DXd enables a decrease in hydrophobicity and helps to increase its DAR, with high stability in plasma. The achievement of a DAR of 8, as well as of the “bystander effect,” gives T-DXd potent antitumor activity in cancer cells with both high and low HER2 expression. These advantages improve also its ability to overcome T-DM1 resistance.^5,43^

Trastuzumab deruxtecan has received the Food and Drug Administration (FDA) and European Medical Agency (EMA) approval for patients with HER2-positive, unresectable, or metastatic breast cancer (DESTINY-Breast03 trial).^44^ Furthermore, it has been observed that ADCs exhibit anticancer properties even in cases of HER2-low breast cancer, wherein the HER2 antigen is expressed on breast cancer cells without any gene amplification. The efficacy of T-DXd in HER2-low breast cancer suggests that ADCs exploit their activity by using the cell antigens as entry points to deliver payloads. In this regard, nearly 60% of breast cancer patients are diagnosed with HER2-low expression, defined as HER2 1+ or HER2 2+ by immunohistochemistry (IHC) and no gene amplification at reflex molecular testing.^45^ DESTINY-Breast04 proved that targeting low level of HER2 with T-DXd was a superior therapeutic approach to standard chemotherapy in patients with unresectable or metastatic HER2-low breast cancer after prior treatment in the metastatic setting.^46^ Based on DESTINY-Breast04, T-DXd received FDA and EMA approval for advanced, pre-treated HER2-low, breast cancer.

Given its novel engineering and in line with the success rates in the metastatic setting, T-DXd is expected to have a substantial impact on the treatment of early breast cancer. Several trials are investigating new treatment protocols for T-DXd in the early breast cancer setting.

In the post-neoadjuvant setting, the randomized, multicenter phase III trial DESTINY-Breast05 (NCT04622319) is evaluating T-DXd vs T-DM1 as a post-neoadjuvant treatment for patients with HER2-positive breast cancer with residual invasive disease post-neoadjuvant therapy. High-risk patients are defined as those with inoperable disease at presentation or with positive pathological axillary lymph node status after neoadjuvant therapy. The study will compare iDFS between the T-DXd and T-DM1 treatment arms. The secondary endpoint is disease-free survival. Based on the results of the DESTINY-Breast03 trial, this study aims to provide benefit in the adjuvant treatment for the same setting as the KATHERINE trial.^28^

On the other hand, the DESTINY-Breast11 trial (NCT051132510) is evaluating T-DXd in the neoadjuvant setting in high-risk, HER2-positive, early non-metastatic breast cancer. Patients are randomized to receive T-DXd monotherapy or T-DXd followed by paclitaxel/trastuzumab/pertuzumab (THP) or dose dense doxorubicin plus cyclophosphamide followed by THP. The trial aims to demonstrate that T-DXd-based regimens are superior to standard treatments by reducing toxicities and improving outcomes.^47^ Similarly, the ADAPT-HER2-trial (NCT05704829) evaluates de-escalation of neoadjuvant therapy in the low-intermediate risk group, testing T-DXd therapy vs standard chemotherapy + trastuzumab + pertuzumab in HER2-positive, low-intermediate risk, node-negative patients with cT1-2 tumors.^48^ Of note, the SHAMROCK trial (NCT05710666) is enrolling patients with HER2-positive early-stage breast cancer to receive six cycles of neoadjuvant T-DXd monotherapy. The primary outcome will be the percentage of patients who achieve a pCR after a T-DXd treatment.^49^ Also, the ARIADNE trial (NCT05900206) is comparing T-DXd to standard preoperative treatment for three cycles in patients with non-metastatic HER2-positive breast cancer. Following initial treatment, patients will receive a further three cycles of therapy based on their intrinsic molecular subtype (PAM50).^50^

Clinical trials are also moving in the early setting of HER2-low breast cancer. In particular, the TRIO-US B-12 TALENT trial (NCT04553770) evaluates T-DXd in the neoadjuvant setting for patients with HER2-low disease. T-DXd is given alone or in combination with ET to patients with HER2-low, HR-positive early breast cancer. Some preliminary results were presented at the San Antonio Breast Cancer Symposium in 2022. Between September 2020 and October 2022, 29 patients were enrolled in each arm and received six cycles of either T-DXd or T-DXd plus anastrozole. The objective response rate was 68% with T-DXd alone and 58% with the combination of T-DXd plus anastrozole. There were two complete responses in each arm; the trial is ongoing and will reveal the role of ET added to an ADC, and the potential role in the curative setting.^51,52^ Also, the TRUDI trial (NCT05795101) investigates the use of T-DXd in combination with immunotherapy. Combination therapy is given as a neoadjuvant treatment for stage III patients with HER2-positive or HER2-low inflammatory breast cancer. The main measure of interest is pCR.^53^

T-DXd appears to be the optimal candidate to implement alternatives for de-escalated treatment options due to its more favorable safety profile than standard anthracyclines and taxane chemotherapy regimens, such as reduced rates of alopecia or polyneuropathy. However, T-DXd has been associated with a high rate of toxicities, such as interstitial lung disease (ILD) that must be carefully identified and timely managed. The median time to onset of ILD appears to be 6 months that is compatible with the usual duration of more intensive regimens in the early setting of breast cancer.^54^ Close monitoring of the patients regarding symptoms of lung disease may be helpful during therapy.

#### Trastuzumab duocarmazine

It (trastuzumab valine-citrulline-seco-duocarmycin-hydroxybenzamide-azaindole [vc-seco-DUBA]; SYD985) is a HER2-targeting ADC, consisting of a humanized anti-HER2 IgG1 antibody, with the same amino acid sequence as trastuzumab, conjugated to a highly potent DNA-alkylating payload, duocarmycin seco-duocarmycin-hydroxy-benzamide-azaindole (a duocarmycin prodrug) via a protease-cleavable vc linker (vc-seco-DUBA) with an average DAR of 2.8 and a cell permeable DUBA that can lead to a bystander effect.^55,56^ Preclinical studies suggest significant activity in HER2-positive breast cancer models, including those with lower HER2-expression. Trastuzumab duocarmazine has already shown promising results in the metastatic setting in the TULIP trial, in the space of HER2-positive cancer.^57^

In the early setting, SYD985 has been selected as part of an investigational treatment arm in the ongoing I-SPY 2 trial (NCT01042379) for patients with HER2-positive and HER2-low early breast cancer. It will be administered as a neoadjuvant treatment to understand the impact on pCR that is the primary endpoint of the study.^58,59^

These ADCs has been associated with ocular toxicities, such as conjunctivitis and keratitis, that can be severe and fatigue.^57^

#### Trastuzumab rezetecan

Trastuzumab rezetecan (SHR-A1811) is an ADC composed of trastuzumab, a stable and cleavable linker, and a novel TopI inhibitor payload (SHR9265) derived from exatecan with a better liposolubility and cellular permeability.^60^ The payload exerts its cytotoxic activity through inhibition of tumor cell proliferation and induction of apoptosis in HER2-expressing tumor cells.^61^ Trastuzumab rezetecan has a DAR of 5.7 and a bystander killing system with good stability and improved safety profiles.^62^ It is currently being evaluated in several clinical trials in patients with HER2-expressing advanced solid tumors, including HER2-positive breast cancer, in both the metastatic and early settings. A phase I study in HER2-expressing or -mutated solid tumors revealed that trastuzumab rezetecan had a favorable antitumor effect with a manageable toxicity profile.^63^

In the early setting, NCT06126640 compares trastuzumab rezetecan to T-DM1, in patients with HER2-positive primary breast cancer with residual invasive disease after neoadjuvant therapy. The primary objective of the study is iDFS.^64^ Trastuzumab rezetecan is also being evaluated as a neoadjuvant treatment in the prospective, single-arm study (NCT05911958) in patients with early or locally advanced breast cancer who are HR-positive and have low HER2 expression.^65^ Finally, the same drug is being evaluated in a single-arm, phase II trial (NCT05635487) in combination with pyrotinib maleate, an irreversible inhibitor of HER1, HER2, and HER4 tyrosine kinases. The combination treatment will be given as neoadjuvant therapy for patients with stage II to III HER2-positive breast cancer. The primary endpoint of the study is the pCR.^66^

#### Disitamab vedotin

Disitamab vedotin (RC48) is an innovative anti-HER2 ADC drug consisting of hertuzumab attached to monomethyl auristatin E (MMAE) via a cleavable linker that is approved for HER2-positive breast cancer in China.^67^ Disitamab vedotin has certain advantages over T-DM1, with good tolerability and good efficacy, even in patients with HER2-low expression.

There are two active clinical trials involving disitamab vedotin in early breast cancer. The NCT05134519 is a phase II clinical trial designed to assess the non-inferiority of disitamab vedotin vs T-DM1 in the context of the neoadjuvant therapy. The primary outcome of the study measures pCR for patients with HER2-positive breast cancer.^68^ Disitamab vedotin is also being evaluated in combination with the PD-1 blocker penpulimab (AK 105) in NCT05726175 trial. This is a phase II clinical trial evaluating the efficacy of disitamab vedotin combined with penpulimab as neoadjuvant therapy for locally advanced breast cancer with HER2-low expression. The primary outcome of the study is pCR.^69^

#### ARX-788

ARX-788 is a new HER2-targeted mAb that is linked to the cytotoxic payload AS269. ARX-788 uses a synthetic amino acids para-acetylphenylalanine (pAF) that is incorporated into a predetermined site on the heavy chain of the monoclonal anti-HER2 antibody and allows for conjugation of a diverse array of payloads. ARX-788 demonstrated activities in HER2-positive, HER2-low, and T-DM1-resistant tumors in preclinical studies.^70^

As neoadjuvant therapy in HER2+ early-stage breast cancer, ARX-788 alone or in combination with PD1 inhibitor is being evaluated in the I-SPY2 trial (NCT01042379).^58^ ARX788 has shown activity, including in some patients following T-DXd, and is being tested in combination with pyrotinib maleate as neoadjuvant treatment in a phase II or III clinical trials NCT05426486 and NCT04983121.^60^

The high stability of ARX-788 and the low serum exposure of pAF-AS269 may underlie low systemic toxicity that differentiates it from other ADCs.^71,72^

#### TQB2102

TQB2102 is an ADC comprised of a humanized antibody against HER2, an enzyme-cleavable linker, and a TopI inhibitor payload, which combine the ability of antibodies to specifically target tumor cells with the highly potent killing activity of drugs with payloads too toxic for systemic administration.^73^

The phase II study (NCT06198751) is evaluating the efficacy and safety of neoadjuvant treatment with TQB2102 for injection in patients with HER2-positive breast cancer.^74^

### Trophoblast cell surface antigen-2-targeting ADCs

Trophoblast cell surface antigen 2 (Trop-2) is a surface glycoprotein expressed on many epithelial tumors.^75^ It is overexpressed in all the subtypes of breast cancer, particularly in more than 85% of triple-negative breast cancer (TNBC) cases, and has been shown to be an unfavorable prognostic factor.^76-79^ Antibody–drug conjugates targeting Trop-2 use mAbs specifically designed to recognize and bind to Trop-2 receptors on cancer cells.^79^ Sacituzumab govitecan and datopotamab deruxtecan (Dato-DXd) are currently the two anti-Trop-2 ADCs being actively studied in breast cancer, and among them sacituzumab govitecan (SG) is only one with preliminary data in early breast cancer.^80^

#### Sacituzumab govitecan

Sacituzumab govitecan consists of the humanized anti-Trop-2 mAb hRS7-IgG1κ and a cleavable linker CL2A conjugated to an active metabolite of irinotecan, SN38. SN38 inhibits TopI and causes cell death.^75,80,81^

In April 2020, SG received accelerated approval for patients with metastatic TNBC who have received at least two prior therapies for metastatic disease (ASCENT trial).^82^ Subsequently, in February 2023 the FDA approved SG for patients with unresectable locally advanced or metastatic HR-positive, HER2-negative (IHC 0, IHC 1+, or IHC 2+/in situ hybridization negative [ISH-]) breast cancer who have received endocrine-based therapy and at least two prior systemic therapies in the metastatic setting based on the TROPiCS-02 trial.^83,84^

SASCIA (NCT04595565) is a phase III trial in HER2-negative breast cancer patients with RD after neoadjuvant chemotherapy. Based on the results of the ASCENT^46^ and TROPiCS 2^82,83^ trials, SG has been tested in the post-neoadjuvant setting in both TNBC and HR-positive/HER2-negative disease. Patients are randomly assigned in a 1:1 ratio to receive SG or treatment of physician’s choice (TPC; either capecitabine, platinum-based chemotherapy, or observation). Patients with HR-positive breast cancer will receive ET according to local guidelines. Adjuvant pembrolizumab will be given until the completion of radiotherapy, before randomization. The primary endpoint will be iDFS. The interim safety analysis presented in the European Society for Medical Oncology (ESMO) Breast cancer congress 2022 revealed that SG showed a higher rate of AEs compared with TPC. Adverse events, especially grades 3 and 4 AE rates were in line with the known safety profile of SG.^85,86^ The COGNITION-GUIDE study (NCT05332561) is a seven-arm umbrella phase II trial designed to evaluate post-neoadjuvant genomic-guided therapy in patients with residual cancer burden (RCB). Patients with Trop-2 overexpression will receive SG.^87^

Combination regimens are also under investigation. ASPRIA trial (NCT04434040) is an ongoing single-arm phase II trial in patients with TNBC and RD after neo-adjuvant therapy. Residual disease is defined as the persistence of cancer in the breast or lymph nodes or positive circulating tumor DNA in the blood. Patients will receive atezolizumab plus SG. The primary endpoint is the rate of undetectable circulating tumor cell-free DNA after six cycles.^88^ Sacituzumab govitecan is also being tested with an anti-PD-1 mAb, pembrolizumab, in three trials: ASCENT-05 (NCT05633654), NeoSTAR (NCT04230109), and NCT05675579. ASCENT-05 is a phase III clinical study. Patients with TNBC will receive SG in combination with pembrolizumab after neoadjuvant therapy and surgery for RD. The study treatment arms include SG plus pembrolizumab, either pembrolizumab, or pembrolizumab plus capecitabine. The primary endpoint is iDFS.^89^ NeoSTAR is a phase II clinical trial evaluating neoadjuvant SG for patients with localized TNBC with tumor size ⩾1 cm, or any size if node positive. Sacituzumab govitecan is administered as neoadjuvant therapy. Following the completion of enrolment in the monotherapy cohort, the combination therapy cohort with SG and pembrolizumab will be opened. The primary objective is to assess pCR. After four cycles, patients with biopsy-proven RD will have the option to receive additional neoadjuvant chemotherapy. Some results from the trial were presented at the 2022 American Society of Clinical Oncology Annual Meeting.^90^ Fifty patients were enrolled in the study, including 26 patients who went directly to surgery after SG. The pCR rate with SG alone was 30% (n = 15; 95% CI: 18%, 45%). Of the 24 patients who received additional neoadjuvant therapy after SG, 6 achieved pCR. A point value should be assigned to BRCA mutation carriers. Eight patients had a germline BRCA mutation. Among them, seven underwent surgery after SG, and six had a pCR (86%, 95% CI 42%, 99%). Patients with BRCA mutation may be particularly sensitive to SG due to the impact of BRCA mutations on DNA repair mechanisms which in fact appears to affect the sensitivity to irinotecan and related compounds.^91^ Finally, NCT05675579 is a phase II trial with the primary objective of evaluating the efficacy of SG and pembrolizumab as a neoadjuvant combination treatment on pCR/RCB-1 in early-stage TNBC in patients who are resistant to combination with immunochemotherapy. The study is ongoing.^92^

The most common AEs with SG are commonly nausea, diarrhea, fatigue, alopecia, neutropenia, anemia, and skin rash. The rates of discontinuation due to AEs were 5.0% in the ASCENT study in metastatic breast cancer, and 11.0% in the SASCIA study in early breast cancer.^82,90^ The genetic polymorphism UGT1*28/28 is involved in the metabolism of SN38 and is associated with a higher risk of AEs, particularly neutropenia.^93^ UGT1A1 genotyping or a primary prophylactic use of granulocyte colony-stimulating factor could be considered in some cases.

#### Datopotamab deruxtecan

Datopotamab deruxtecan consists of a humanized IgG1 mAb targeting TROP2 attached via a cleavable linker to a TopI inhibitor payload.^80^ In the TROPION-PanTumor01 study (NCT03401385), patients with heavily pretreated TNBC who were treatment naïve to TopI inhibitor-based therapies had an overall response rate (ORR) of 52%. Such results are leading to further studies in both metastatic and early breast cancer.

The TROPION-Breast03 trial (NCT05629585) is an ongoing study of adjuvant therapy in patients with RD after neoadjuvant systemic therapy. It is a phase III, open-label, randomized trial assessing the efficacy of Dato-DXd with or without durvalumab when compared with capecitabine and/or pembrolizumab in participants with stage I to III TNBC. The primary endpoint of the study is iDFS.^94^

### HER3-targeting ADCs

HER3 is a member of the human epidermal growth factor receptor family that mediates resistance to HER2 and PI3K pathways directed therapies via protein kinase B (AKT)signaling.95

#### Patritumab deruxtecan

Patritumab deruxtecan (HER3-DXd; U3-1402) is constructed by a fully human anti-HER3 IgG1 mAb linked to a TopI inhibitor payload (MAAA-1181a), via a tetrapeptide-cleavable linker.

The TOT-HER3 (NCT04610528) is a clinical trial designed to evaluate the biological activity in treatment-naïve patients with HR-positive/HER2-negative disease. The primary tumor must be ⩾1 cm. Patients are enrolled in four cohorts according to mRNA-based ERBB3 expression. The primary endpoint is the CelTIL score (a biomarker combining tumor cellularity and tumor-infiltrating lymphocytes) after a single dose of patritumab deruxtecan. Data from part A of the TOT-HER3 trial revealed that HER3-DXd is associated with clinical response, increased immune infiltration, and proliferation suppression with an acceptable safety profile in patients with HR+/HER2-negative early breast cancer, regardless of baseline ERBB3 levels. In part A, a significant change in CelTIL score was observed, with a median increase from baseline of 3.5 (interquartile range, −3.8 to 12.7; *P* = .003). In terms of clinical response, an ORR of 45% was observed in 62 patients, with a trend toward an increase in CelTIL score among responders compared with non-responders with a mean difference (+11.9 vs +1.9). The change in CelTIL score observed was independent of baseline ERBB3 mRNA and HER3 protein levels. There were genomic changes, including switching toward a less proliferative tumor phenotype based on PAM50 subtypes, suppression of cell proliferation genes, and induction of genes associated with immunity.^96-98^ Data from the part B presented at ESMO 2023 showed a statistically significant change in tumor cellularity and tumor-infiltrating lymphocyte (CelTIL) score overall (*P* = .046). The ORR was 32% (35% in TNBC and 30% in HR+/HER2−).^97^ Another study, the VALENTINE trial (NCT05569811), is testing chemotherapy or patritumab deruxtecan in early HR-positive, HER2-negative, and high-risk breast cancer. This study aims at evaluating the clinical benefit and biological effects of HER3-DXd with/without letrozole as a neoadjuvant treatment regimen.^99^ Results have not been reported.

## LIV1-Targeting ADCs

LIV-1 is a cell surface membrane protein that is upregulated in ER-positive breast cancer and also in TNBC.^100^

### Ladiratuzumab vedotin

Ladiratuzumab vedotin (SGN-LIV1A) is an ADC that targets LIV-1 composed of a humanized mAb, IgG1, connected through a cleavable linker to a cytotoxic agent, MMAE.^100^ Once internalized, it releases its cytotoxic payload by proteolysis in the lysosomes. The cytotoxic payload is a potent microtubule-disrupting agent.100

The I-SPY (NCT01042379) is an adaptive clinical trial platform evaluating the efficacy of ladiratuzumab vedotin as part of the neoadjuvant treatment of high-risk early breast cancer. Ladiratuzumb vedotin is followed by doxorubicin/cyclophosphamide (AC). The control arm will receive weekly paclitaxel followed by AC. Pathological complete response is the primary endpoint. In the first report provided, ladiratuzumab vedotin did not improve pathological responses compared to the control arm.^101^

## Challenges and Strategies to Overcome Resistance Mechanisms in ADCs

**Figure 3.**
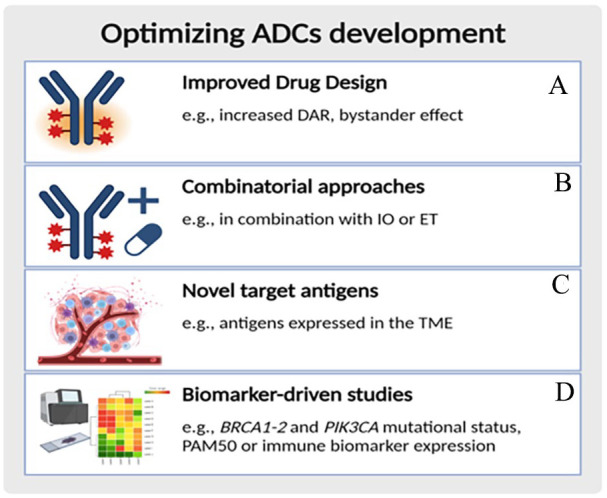
Optimizing ADC development. (A) High DAR and strong bystander effects may potentiate the efficacy of the new generation ADCs against heterogeneous tumors or to those with lower target expression. (B) Choosing the most appropriate ADC drug with the most suitable combination partner. (C) Components of the neovascular system or the tumor matrix could represent target antigens with a more stable genome, reducing the mutational escape of cancers. (D) Biomarker-enhanced studies may better portray sensitivity and resistance to ADCs. Genomic profiling for luminal A tumors (by PAM50), PIK3CA control, BRCA mutation status, and immune marker expression could refine the outcomes of systemic de-escalated targeted versions. ADC indicates antibody–drug conjugate; *BRCA 1-2*, BReast CAncer gene 1-2; DAR, drug-to-antibody ratio; ET, endocrine therapy; IO, immunotherapy; *PIK3CA*, Phosphatidylinositol-4,5-Bisphosphate 3-Kinase Catalytic Subunit Alpha; TME, tumor microenvironment. Created with biorender.com (license, IEO).

Despite the important progress of the new generation of ADCs, challenges remain and strategies are still needed to optimize the use of ADCs in cancer treatment (Figure 3).

Challenges with ADCs certainly include AEs, which may be due to a variety of factors such as target selection, drug mechanisms, chemical properties of linkers, and binding sites.^102^ The immune response can be stimulated by ADC drugs, which can lead to reactions, resulting in AEs such as ILD.^103,104^

Drug efficiency poses a second significant challenge specific to ADCs. A suitable half-life ensures that ADC can reach tumors, and the strength of the linker prevents premature release of the cytotoxic payloads.^105^ Internalization is also important to display drug efficacy.^106^ Moreover, the expression rate of the antigens and the molecular weight of ADCs need to be determined. Given the fact that only a small fraction of the ADCs can effectively reach the tumor site, the toxicity of payloads should be potent while avoiding toxicity. An additional challenge is drug resistance. It can be both primary (initially resistant) and secondary (resistant after response). Reasons for drug resistance can be antibody related, payload related, and cancer cell function related.^107^ Inadequate antigen expression levels can lead to loss of ADC specificity, and mutated payload targets can also lead to treatment failure.^108^ In cancer cells, impaired trafficking and disrupted lysosomal function contribute to drug resistance.

Biomarker-enhanced studies may better portray sensitivity and resistance to ADCs. Genomic profiling for luminal A tumors (by PAM50), PIK3CA mutation, BRCA status, and immune marker expression could refine the outcomes of systemic de-escalated treatments.^32^ The identification of new targets with tumor-specific expression, such as Trop-2, LIV-1, and HER3, or improved conjugation technologies to optimize payload/release are certainly of paramount importance to improve ADC therapy and catalyze their development toward early breast cancer. High DAR and strong bystander effects may potentiate the efficacy of the new-generation ADCs against heterogeneous tumors or to those with low target expression.^5,6,20^

As a future perspective, components of the neovascular system or the tumor matrix could represent target antigens with a more stable genome, reducing the mutational escape of cancers.^8^

Among the mechanisms to overcome resistance to ADCs is the concept of dual-epitope or dual-target ADCs that has inspired ADC innovations. Bispecific antibody–drug conjugates are a promising candidate in the field of targeted cancer therapy.^109,110^ By simultaneously targeting two different antigens, compounds increase tumor cell specificity and reduce toxicity to normal tissues. In addition, the dual-targeting approach can overcome drug resistance caused by decreased expression of a single target, offering the potential for improved treatment outcomes in patients with resistant cancers.

The limited availability of targets in breast cancer, such as HER2 and HER3, has limited the rapid development of these molecules. Bispecific antibody–drug conjugates, such as zanidatamab zovodotin (ZW49), are under development (NCT03821233). Combination therapies with ADCs offer a strong biological rationale to overcome resistance. Antibody–drug conjugates could be combined with immunotherapy, TKIs, endocrine therapies, and mAbs.^34^ In combination with immunotherapy, ADCs can trigger mechanisms like immunogenic cell death and T cell infiltration, while immune-checkpoint inhibitors reinvigorate exhausted T cells, leading to potential synergistic effects.

## Conclusions

In early breast cancer, at least seven ADCs targeting HER2, HER3, TROP2, or LIV1 are currently under investigation in various phases of clinical trials. Of these, T-DM1, T-DXd, and SG are the ADCs at a more advanced stage of development.

New ADCs under development are also promising and have shown good results such as ARX788 and TQB2102.

Imminent and important results are expected from studies evaluating ADCs as monotherapy or in combination with immunotherapy or ET in HER2-positive, HER2-low, and HER2 0 early breast cancer, either HR-positive or TNBC type. Many of the ADCs are being developed in combination strategies, as add-ons or in place of standard treatments. De-escalation is the main treatment goal in most of these trials.^32^

There remains an urgent need for strategic approaches to choosing the most appropriate ADC drug and ultimately the most suitable combination partner. The goal should be to achieve a higher cure rate with the lowest rate of side effects. A key component must be the optimal patient selection, based on the predictive biomarkers, including cancer genetic profiles, tumor’s targetable component, and clinical biomarkers.

Hopefully, at the end of the journey, *Ithaka will be reached.*
